# A neonatal presentation of factor V deficiency: A case report

**DOI:** 10.1186/1471-2431-7-8

**Published:** 2007-02-21

**Authors:** Amol Chingale, Michael Eisenhut, Anjali Gadiraju, Ri Liesner

**Affiliations:** 1 Department of Pediatrics, Luton and Dunstable Hospital NHS Foundation Trust, Luton, Bedfordshire, LU 4 0DZ, UK; 2 Department of Hematology, Great Ormond Street Hospital for Children, London, WC1N 3JH, UK

## Abstract

**Background:**

Factor V deficiency is a rare autosomal recessive coagulation disorder. Awareness of presenting features and management is important to avoid bleeding complications associated with mortality and neurodisability.

**Case presentation:**

A 6-day-old Pakistani boy was admitted with bleeding from the left nipple. His parents were first cousins. A coagulation screen showed a prothrombin time of 41 s (control 14 s), a partial thromboplastin time of 132 s (control 33 s) and a normal thrombin time of 15 s (control 14 s). Factor V activity was <0.01 IU/ml. Oral tranexamic acid was started. At 5 weeks of age the child presented with irritability, lethargy and reduced feeding and a drop of hemoglobin to 5.6 g/dl. A cranial computed tomography scan showed a right intra-cerebral bleed extending from the frontal lobe to the parieto-occipital region with shift of the midline to the left. A regime of 20 ml/kg of fresh frozen plasma four times a week was instituted and has prevented further bleeds up to the present age of 21 months. Neurodevelopment remained normal.

**Conclusion:**

This case illustrates that in an unusually bleeding newborn of consanguineous parents rare severe homozygous bleeding disorders need to be considered. Nipple bleeding may be the first presentation of a congenital bleeding disorder. In cases of factor V deficiency where factor concentrates are not available long term use of fresh frozen plasma can prevent potentially life threatening bleeding.

## Background

Congenital deficiency of factor V (labile factor, proaccelerin) is a rare hereditary coagulation disorder with an incidence of 1:1000,000 and is inherited as an autosomal recessive trait [1]. The active form of coagulation factor V is involved as an essential non-enzymatic cofactor for the activated factor X catalysed conversion of prothrombin to thrombin. Affected patients become symptomatic in early childhood with spontaneous or post-traumatic bleeding complications [2]. We present to our knowledge the first case of nipple bleeding as the initial hemorrhagic manifestation of factor V deficiency in the neonatal period. It preceded an intracerebral hemorrhage.

## Case presentation

A 6-day-old Pakistani boy was admitted to hospital in June 2005 for bleeding from the left nipple. His parents are first cousins. He has two siblings aged 8 and 6 years. There was no family history of bleeding diathesis. The mother had a normal pregnancy with full antenatal care. Mode of delivery at term was an elective Caesarean section due to previous Caesarean sections. His birth weight was 2.7 kg. 1 mg of Vitamin K was given intramuscularly at delivery. His first neonatal check was normal. He had a small amount of self-limiting bleeding from the umbilical cord on day 3 of life. He went home on day 4.

On day 6 of life he had mild spontaneous left sided nipple bleeding, which presented as spots of blood stains on the left side of his baby dress.

Over the next 7 days he had two further episodes of a similar nature. Baseline biochemistry and full blood count were normal but a coagulation screen showed a prolonged prothrombin time of 41 s (control 14 s), a prolonged partial thromboplastin time of 132 s (control 33 s) and normal thrombin time of 15 s (control 14 s). In view of the mild nature of bleeding, the child was only treated with 1 mg of i.v. vitamin K. Subsequent coagulation assays revealed a plasma factor V activity of less than 0.01 IU/ml (normal range 0.50–1.50 IU/ml) determined by factor V clotting assay with all other coagulation factors in the normal range. Hence the diagnosis of congenital factor V deficiency was made. A cerebral ultrasound done at this stage was within normal limits. Oral transexamic acid at a dose of 15 mg/kg/dose 3 times daily was started.

Mother was found to have a plasma factor V activity of 0.44 IU/ml (normal range 0.5–1.50 IU/ml) and the father of 0.52 IU/ml by factor V clotting assay. Both levels are compatible with heterozygous factor V deficiency. The siblings had factor V activities of 0.61 and 1.1 IU/ml respectively. Genetic analysis at the St. Thomas Hospital hemophilia molecular genetics laboratory showed that the affected child is homozygous for a frame shift mutation resulting in a premature termination sequence at codon 2178 in exon 25 of the factor V gene and both parents are heterozygous for this mutation.

The infant presented at day 15 of age with a further episode of bleeding from the umbilical stump. The bleeding stopped promptly following administration of 20 ml/kg of fresh frozen plasma (FFP, methyleneblue sterilized, single donor and US sourced). Small bruises were noted around the venepuncture sites from the previous admission. A hematoma measuring 2 cm in diameter was noted on the anterolateral aspect of the left thigh. It was attributed to the injection of vitamin K at birth. His head circumference was increasing within normal range and a repeat cerebral ultrasound was normal.

**Figure 1:**
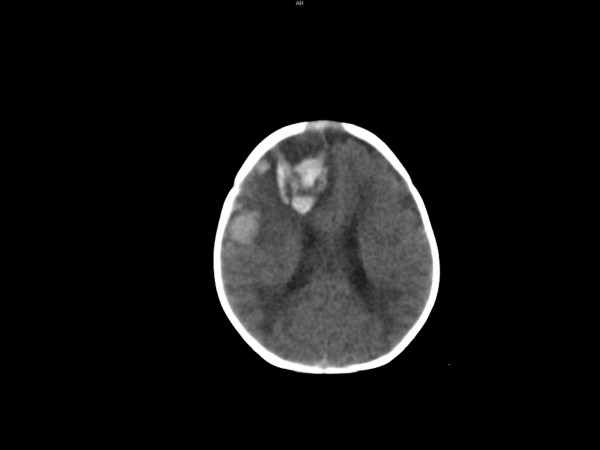
Computed tomography of the brain conducted at 5 weeks of age showing an intra cerebral hemorrhage.

The patient presented again at 5 weeks of age with pallor, irritability, lethargy and reduced feeding. His anterior fontanelle was bulging and tense. He was hemodynamically stable. His hemoglobin was 5.6 g/dl, which represented a significant drop from the previous estimation 3 weeks back. The cerebral ultrasound was repeated and showed a large intracerebral hemorrhage extending from the frontal lobe to the parieto-occipital region. There were a few cystic areas seen within this bleed, which suggested that the bleeding started at least a few weeks ago. The child was immediately transfused with 20 ml/kg of FFP and 15 ml/kg of packed red blood cells. He had a series of generalized fits within 5 hours of admission. The patient was transferred to the regional tertiary referral centre for specialised neurosurgical and haematological management. A cranial computed tomography (CT) scan showed a right, large, intracerebral bleed causing a shift of the midline to the left. The ventricles were not enlarged and the bleed did not extend into the ventricles (See Figure 1). A Hickman line insertion was arranged to enable regular FFP infusions. Preoperatively he was transfused again with 20 ml/kg FFP and 10 ml/kg of platelet concentrate to achieve adequate hemostasis. Platelets are known to be a good source of factor V as it is stored in the platelet alpha granules. He was also given recombinant factor VIIa (Novoseven, NovoNordisk^®^) as an empirical adjunctive agent in the peri-operative period in view of the fact that reliably hemostatic levels of factor V (0.25–0.30 IU/ml) are difficult to achieve in this condition. Factor VIIa is unlikely to work in the absence of any factor V but can contribute to increased thrombin formation once factor V is present. Postoperatively a regime of FFP (15 ml/kg/dose) administration was set up: FFP twice a day for the first 10 days followed by once a day for the next 10 days. This was followed by at least alternate days (every 48 hours) of FFP at 20 ml/kg/day up to the present. Plasma factor V activity has been monitored: A maximum factor V level of 0.23 IU/ml was achieved within 20 minutes after transfusion. Trough levels were between <0.01 to 0.05 IU/ml. When undetectable factor V levels were noted inhibitor assays were performed. Inhibitors have not been detected so far. No further bleeding has been noted up to the present day. Immunisations including hepatitis B vaccination were given subcutaneously as is normal practice for children with severe bleeding disorders. Follow up until the present (21 months of age) revealed an appropriate increment in head circumference. Neurodevelopment has been within normal limits.

## Conclusion

Our report describes the 6^th ^case of homozygous severe factor V deficiency presenting as bleeding in the early neonatal period reported in the English literature. Clinical and laboratory features of the other cases [1-5] are listed in Table 1. Unilateral nipple bleeding as the first significant bleeding manifestation has not been reported in a coagulation disorder before. The differential diagnosis of nipple bleeding in infancy includes trauma, infection or spontaneous bleeding of a hypertrophic (due to maternal hormones like estrogen, progesterone and prolactin) breast gland [6], papillomas within the breast gland [7] and infantile cystic ductal hyperplasia of the breast gland [8]. In our case there were no clinical features supporting any of these diagnoses. Mild hemorrhagic manifestations of coagulation disorders in infants reported previously included umbilical stump bleeding, subcutaneous hematomas, epistaxis and gum bleeding [9]. This case illustrates that in an unusually bleeding newborn of consanguineous parents rare severe homozygous bleeding disorders need to be considered. The mild first manifestations need to alert the clinician and lead to the prompt initiation of coagulation studies bearing in mind that in factor XIII deficiency coagulation tests are normal and factor analysis is required. Even though factor V deficiency can be recognized early prophylaxis of bleeding complications like intracerebral bleeds is difficult in the absence of a factor concentrate and in view of the fact that the risk of intracerebral bleeds for the individual patient is unknown. Intracerebral or subdural bleeds are the most feared complication and have been noted in about 1/10 reported cases in the neonatal period which may be an overestimate due to overreporting [4]. In our case, a life threatening intracerebral bleed occurred at 5 weeks of age. Though rare, congenital factor V deficiency should be kept in mind as a differential diagnosis in an otherwise well term infant presenting with catastrophic intracranial bleed, particularly if the parents are consanguineous [2].

Prolonged PT and PTT with normal thrombin time and normal platelet count would be an initial pointer towards an inherited disorder of coagulation. Clotting factor assay revealing markedly reduced plasma factor V activity confirms the diagnosis [4]. As there is no specific concentrate available, the mainstay of treatment for severe factor V deficiency is FFP. Platelets also contain factor V, though their use should be reserved for life threatening bleeding and prior to surgery. There is a considerable variation in the volume of FFP and frequency of its administration needed to achieve hemostasis for a particular bleeding episode in the individual patient [4,10]. FFP administration should be titrated according to estimated changes in the plasma factor V activity [5]. The half-life of factor V is 12–36 hours and is probably lower in neonates. Therefore regular infusions at least every 48 hours are required in patients with a history of severe bleeding complications. Despite such a regime it is difficult to achieve a factor V level >0.3 IU/ml without encountering problems of fluid overload because of the fact that FFP is not a concentrated treatment.

In our patient one could argue that early institution of regular FFP infusions could have prevented the intracranial hemorrhage but it is crucial to establish in each case that they have a severe bleeding phenotype before embarking on regular FFP infusions, which carry the risks associated with central venous access and FFP and platelet transfusion. Many reported cases of severe factor V deficiency did not have such severe bleeds and only needed treatment at times of surgical challenge [11,12]. At present it is difficult to predict the clinical course with regards to the risk of severe bleeding complications. Future genetic studies may improve our understanding of the risk of developing a severe phenotype associated with a specific genotype. As severe factor V deficiency with a clinically severe phenotype is a very rare disorder of hemostasis the long term prognosis cannot be delineated. It is very likely that cases such as this did not survive infancy in the past and even now the prognosis must be guarded. Detection of the causative mutation is important as it facilitates genetic counselling of the parents and prenatal diagnosis in future pregnancies.

## Competing interests

The authors declare that they have no competing interests.

## Authors' contributions

AC, ME and AG participated in conception and design of this report and were involved in drafting the manuscript and revising it. RL participated in acquisition of data and critical revision of the manuscript. All authors read and approved the final manuscript.

## Pre-publication history

The pre-publication history for this paper can be accessed here:



## Figures and Tables

**Table 1:** Cases of neonatal presentations of factor V deficiency reported in the English literature so far.

Age at first presentation	Birth	4 Days	10 days	18 Days	Birth
Clinical presentation	Hydrocephalus secondary to unilateral subdural hematoma	Large left subdural hematoma causing irritability, pallor, poor feeding	Antenatal diagnosis of increased head circumference and unilateral ventricular dilatation at 32/40, seizure on Day 10	Pallor, excessive crying, bleeding from umbilical stump, Large unilateral intracerebral bleed with intraventricular extension with hydrocephalus	Subdural hematoma
Family history	Consanguineous parents, no bleeding diathesis in family	Nonconsanguineous parents, no bleeding diathesis in family	Nonconsanguineous parents, no bleeding diathesis in family	Consanguineous parents, no bleeding diathesis in family	Nonconsanginous, no bleeding diathesis in family
Clotting screen on presentation	PT: 33 (14) PTT: 87 (33)	PT: Very high PTT: immeasurable	PT: 61 s (31 s) PTT: 178	Prothrombin ratio: 2.56 (Normal 1–1.2) APTT: >200	PT: 58 s(control 8.7–11.5 s), APTT 198.8 s (control: 29.5–42.7 s)
Factor V activity in baby (reference 70–120%)	< 1%	< 5%	2%	3% Presence of Factor 5 inhibitor 2.4 U	<0.4%
Factor V activity in parents	54%, 42%	35%, 40%	52%, 78%	40%, 63%	59%, 47%
Treatment	Virus inactivated FFP	Virus inactivated FFP	FFP	FFP, platelet concentrate, activated prothrombin complex, immunoglobulins, single-volume exchange transfusion	No data
Outcome	Global neurodevelopemental delay	At 6 months, neurologically normal, but recurrent mucosal and soft tissue hemorhages	VA shunt to drain the hydrocephalus	VP shunt for hydrocephalus, death following another episode of intracranial bleed	No data
References	(4)	(2)	(3)	(5)	(1)
